# A Microsphere-Based Sensor for Point-of-Care and Non-Invasive Acetone Detection

**DOI:** 10.3390/bios15070429

**Published:** 2025-07-03

**Authors:** Oscar Osorio Perez, Ngan Anh Nguyen, Landon Denham, Asher Hendricks, Rodrigo E. Dominguez, Eun Ju Jeong, Marcio S. Carvalho, Mateus Lima, Jarrett Eshima, Nanxi Yu, Barbara Smith, Shaopeng Wang, Doina Kulick, Erica Forzani

**Affiliations:** 1 School of Engineering for Matter, Transport and Energy, Arizona State University, Tempe, AZ 85287, USA; oosoriop@asu.edu (O.O.P.); annguye6@asu.edu (N.A.N.); lrdenham@asu.edu (L.D.); ajpete20@asu.edu (A.H.); redoming@asu.edu (R.E.D.); ejeong5@asu.edu (E.J.J.); 2 Center for Bioelectronics and Biosensors, Biodesign Institute, Arizona State University, 1001 S McAllister Ave., Tempe, AZ 85281, USA; nanxiyu@asu.edu (N.Y.); shaopeng.wang@asu.edu (S.W.); 3 Medical Devices and Methods Laboratory, Health Futures Center, Arizona State University, 6161 E. Mayo Blvd., Phoenix, AZ 85054, USA; 4 Department of Mechanical Engineering, Pontifícia Universidade Catolica do Rio de Janeiro, Prédio Pe. Laércio Dias de Moura-R. Marquês de São Vicente, 225-6ºandar–Gávea, Rio de Janeiro 22451-900, Brazil; msc@puc-rio.br (M.S.C.); mlima@lmmp.mec.puc-rio.br (M.L.); 5 School of Biological and Health Systems Engineering, Arizona State University, G Wing, E. Tyler Mall, Tempe, AZ 85281, USA; jeshima@asu.edu (J.E.); barbarasmith@asu.edu (B.S.); 6 Mayo Clinic Arizona, 13208 E. Shea Blvd., Scottsdale, AZ 85259, USA

**Keywords:** acetone sensor, liquid-core microspheres, colorimetric detection, non-invasive monitoring, point-of-care diagnostics, breath and skin acetone, metabolic health, ketosis, obesity, type 1 diabetes

## Abstract

Ketones, which are key biomarkers of fat oxidation, are relevant for metabolic health maintenance and disease development, making continuous monitoring essential. In this study, we introduce a novel colorimetric sensor designed for potential continuous acetone detection in biological fluids. The sensor features a polydimethylsiloxane (PDMS) shell that encapsulates a sensitive and specific liquid-core acetone-sensing probe. The microsphere sensors were characterized by evaluating their size, PDMS shell thickness, colorimetric response, and sensitivity under realistic conditions, including 100% relative humidity (RH) and CO_2_ interference. The microsphere size and sensor sensitivity can be controlled by modifying the fabrication parameters. Critically, the sensor showed high selectivity for acetone detection, with negligible interference from CO_2_ concentrations up to 4%. In addition, the sensor displayed good reproducibility (CV < 5%) and stability under realistic storage conditions (over two weeks at 4 °C). Finally, the accuracy of the microsphere sensor was validated against a gold standard gas chromatography-mass spectrometry (GC-MS) method using simulated and real breath samples from healthy individuals and type 1 diabetes patients. The correlation between the microsphere sensor and GC-MS produced a linear fit with a slope of 0.948 and an adjusted R-squared value of 0.954. Therefore, the liquid-core microsphere-based sensor is a promising platform for acetone body fluid analysis.

## 1. Introduction

Metabolic health, which is essential for overall well-being, is acknowledged as a key factor in health maintenance and disease development [1]. While traditional disease diagnosis often focuses on treating symptoms, a more holistic approach recognizes that metabolic imbalances frequently precede and contribute to disease [2]. Understanding the underlying metabolic changes is fundamental for effective prevention and treatment. An emerging view is that optimal cellular homeostasis is essential for maintaining health. This balance involves not only the right amounts of nutrients but also the efficient processing of energy from diverse sources. Disruptions in these delicate metabolic processes, whether due to deficiencies, excesses, or imbalances in energy flow, as well as variations in energy density within cellular metabolism, can lead to metabolic diseases [3].

Fat metabolism, which is intrinsically related to energy needs, plays a central role in human physiology. Fat represents a variable portion of adult body mass (3 to 42%) [4] and it works alongside carbohydrate metabolism to fuel the body. As described by Randle and colleagues in the 1960s, the “glucose-fatty acid cycle” elegantly illustrates the reciprocal relationship between fat and carbohydrate oxidation in muscle [5]. These two key metabolic pathways are maintained in a delicate biochemical and functional balance, and they are dynamically regulated by factors such as diet, physical activity, hormones, and even mood [4]. Therefore, continuous monitoring of our metabolic status is not only important but also necessary to evaluate, prevent, and treat disorders and diseases. Advancements in science and technology require the development of robust detection methods for continuous, non-invasive metabolic monitoring using reliable biomarkers, enabling timely, point-of-care interventions for improved health outcomes.

Ketone bodies, specifically β-hydroxybutyrate (BHB), acetoacetate (AcAc), and acetone, are produced during fatty acid breakdown and serve as key indicators of a ketogenic state, where the body does not use glucose for energy but instead uses fatty acids [6]. Acetone, primarily formed through the enzymatic decarboxylation of acetoacetic acid (AcAc), has emerged as a promising biomarker for monitoring ketosis. Tracking ketone levels is clinically relevant for managing various health conditions. These include eating disorders such as anorexia and obesity [7], diabetic ketoacidosis (a complication of type 1 diabetes), epilepsy [8], cardiovascular diseases and cholesterol disorders [9], cancer [10], kidney diseases [11], and Parkinson’s disease [12], among others.

Current ketone detection methods range from highly precise laboratory-based techniques, such as gas chromatography-mass spectrometry (GC-MS), high-performance liquid chromatography (HPLC), and selected ion flow tube–mass spectrometry (SIFT-MS) [6,13,14], to point-of-care tests, including enzymatic assays and colorimetric urine reactions [15,16]. While laboratory methods offer high accuracy, their non-portability, requirement for trained personnel, and high cost limit their applicability for continuous or personal monitoring [6]. Point-of-care methods, while more accessible, often suffer from a limited dynamic range and susceptibility to interference from hydration status and food consumption, and they are invasive [15,16]. Thus, the measurement of ketones, particularly acetone, which is a non-invasive biomarker, has not been achieved as a basic practice for health care through monitoring the metabolic state of fat oxidation. Therefore, it is desirable to have a method and device to measure the degree of ketosis accurately, passively, simply, and at a reasonable cost that favors the metabolic monitoring of individuals.

Breath acetone detection devices, which typically employ metal oxide sensors, have become commercially available for point-of-care use [6]. These devices can detect acetone in the parts-per-million (ppm) range, providing users with an estimate of their breath acetone levels. However, concerns remain regarding sensor sensitivity decay, variable sample exhalation rate, humidity interference, and power consumption [17]. Furthermore, the lack of rigorous clinical validation for many of these devices raises questions about their reliability and accuracy [6]. Wearable skin acetone sensors offer a potentially attractive non-invasive monitoring approach; however, they are still in the early stages of development. Current research efforts often involve sample pre-concentration techniques, which complicate real-time monitoring. Critically, the long-term stability of these sensors, which is essential for practical, widespread use, has not been adequately addressed [18,19]. Therefore, a significant need remains for a reliable, non-invasive, cost-effective method for continuous acetone monitoring. Such a method would enable individuals to easily track their metabolic state, thereby facilitating proactive health management and potentially improving the diagnosis and management of various diseases.

Traditional colorimetric acetone detection methods use compounds such as nitroprusside-based or aminopropyl silica materials that exhibit low accuracy and require complicated breath collection and analysis setups, thereby increasing device cost and complexity [20]. In this work, we introduce a novel colorimetric acetone sensor to overcome these limitations, leveraging the high sensitivity and selectivity achieved by incorporating hydroxylamine sulfate (HA) and thymol blue (TB) within PDMS microspheres. This sensor operates based on the selective reaction of ketones with HA, generating protons that induce a visible color change in TB from yellow to pink within a specific pH range (see Section 2.2). The selection of TB was strategic, as its colorimetric response is well-suited to the pH changes that result from the acetone-driven reaction, thereby enabling a simple visual and quantitative detection system. This innovative approach facilitates the creation of a calibrated breath sampling and image capture device, which demonstrated selectivity and has been validated against a reference instrument for ketosis monitoring. This work presents the novel sensor, its current analytical performance, and applications for breath analysis, as well as potential future skin detection using a liquid-core microsphere platform. We aim to develop a stable, selective, and sensitive sensor that is suitable for real-world applications. Two critical challenges have been addressed: preventing the degradation of the sensing probe over time and establishing a reproducible fabrication process. We overcame these challenges by developing a novel acetone sensor based on liquid-core microspheres. The liquid-core design mitigates the degradation of the sensing probe, while the microsphere fabrication method facilitates reproducible liquid encapsulation for mass production.

## 2. Materials and Methods

### 2.1. Materials

Hydroxylamine sulfate (Fluka, Buchs, Switzerland) and thymol blue (Acros Organics, Geel, Belgium) were used for the liquid sensing probe. Polyvinyl alcohol (PVA), glycerol, methanol, and sodium hydroxide were purchased from Sigma Aldrich, St. Louis, MO, USA. High-purity nitrogen (grade 5.0, with a maximum of 10 ppm total impurities, including <1.0 ppm oxygen, <1.0 ppm moisture, and <0.5 ppm hydrocarbons) was used for background measurements and washing bags. For simulated breath sample preparation, 40 L aluminum Tedlar^®^ bags (Custom Sensors Solutions, Oro Valley, AZ, USA) were filled using a certified gas mixture of CO_2_ (4.02% *v*/*v*) and O_2_ (16.10% *v*/*v*) balanced in nitrogen (Matheson™, 1545 Watkins St., Phoenix, AZ, USA), as well as a precision acetone gas mixture (1000 ppm *v*/*v* in nitrogen, GASCO™, Oldsmar, FL, USA). Ultrapure water, produced by an ELGA Purelab Ultra RO system, was used to create 100% relative humidity (RH) in the samples.

### 2.2. Colorimetric Acetone Detection

Acetone detection occurred within an aqueous sensing probe that contained hydroxylamine sulfate (HA) and thymol blue (TB, a pH indicator). Acetone reacted with HA in the presence of TB to form a stable oxime derivative and sulfuric acid. The resulting acid generation caused a localized pH decrease, which was quantified by the colorimetric change in TB (yellow to pink at its first *pKa* of ~2.0) [20]. Because tiny amounts of acid induce a significant color change in TB, this method has been used for environmental acetone detection in exposure assessments [21]. Figure S1 shows the chemical reaction of the colorimetric acetone sensor detection.

### 2.3. Microsphere Fabrication

**Figure 1:**
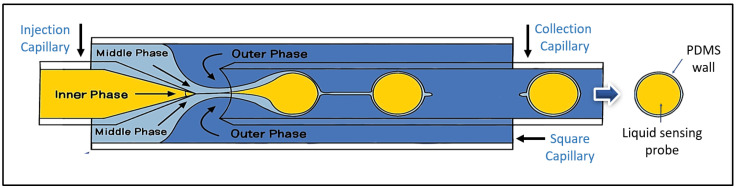
Schematic representation of a liquid-core microsphere showing the encapsulation of a liquid sensing probe by a PDMS shell.

Microfluidic devices were meticulously designed and fabricated for liquid-core microspheres by drawing upon established methodologies from Utada (2005) and Do Nascimento (2017) [22,23]. Details of the setup are presented in the Supplementary Information section. The architecture of the device, as depicted in Figure S2, consisted of two cylindrical glass capillary tubes (VitroCom™, Mountain Lakes, NJ, USA), one ending in a conical tip and the other with a flat tip, coaxially aligned within a square glass capillary tube (VitroCom™), which was integrated onto a standard glass microscope slide. The conical tip was shaped using a micropipette puller (Sutter™, Alpharetta, GA, USA) to ensure flow control. The alignment of the cylindrical capillaries within the square capillary was performed manually under microscopic observation. A microscopic image of the microsphere formation region is presented in Figure S3. Experimentally, minor deviations in this alignment yielded an acceptable microsphere size distribution. Figure S4 presents representative size distributions from multiple devices with comparable alignment errors, demonstrating consistent results. In cases of significant misalignment that impeded the generation of desired homogeneous microspheres, the affected devices were replaced. In addition, two needles for input solutions are connected to the top of the device. A three-phase flow system controlled by three independent pumps (Chemyx™, Stafford, TX, USA) was employed to inject the different solutions into the device, with each pump regulating the flow rate of the inner, middle, and outer phases. The experimental setup for microsphere fabrication, including the microscope and the three pumps for the sensing solution, PDMS, and PVA, is shown in Figure S5. The inner phase, a sensing solution for acetone detection, was prepared by dissolving HA and TB in a methanol–water–glycerol solvent mixture, with the pH adjusted to 5 according to established protocols [20]. The middle phase consisted of polydimethylsiloxane (PDMS, Sylgard^®^ 184, purchased from Sigma Aldrich, St. Louis, MO, USA), following the manufacturer’s specifications without the use of a polymer precursor or a solvent for linker dispensing. The time between mixing the PDMS components and their introduction into the fluidic system was approximately 15 to 30 min. The outer phase consisted of a 10% w/v solution of polyvinyl alcohol (PVA), which facilitated the controlled flow of the medium along the microfluidic device walls. This solution was stored at 4 °C until use. The sensing probe solution (HA-TB) was pumped through the lumen of the conical tip capillary (Figure 1, left side). Simultaneously, a hydrophilic PVA solution (outer phase) was pumped in the opposite direction through the annular space surrounding the flat-tipped capillary (Figure 1, right side). The device was also supplied with a third hydrophobic middle phase solution of PDMS, which was co-injected alongside the conical capillary, flowing in the same direction as the HA-TB solution. (Figure 1, left side). Figure S6 illustrates the microsphere fabrication process as visualized under microscopy. The interaction of these three fluids, driven by differences in hydrophobicity, surface tension, and viscosity, resulted in the formation of three-phase microspheres: a hydrophilic core of HA-TB, a PDMS shell, and an outer layer of PVA. The PVA layer was partially removed via repeated washing with distilled water (5 mL × 3) and dried in an inert atmosphere at 4 °C until use. This optimized fabrication method allowed for the precise control of microsphere formation and its structural characteristics.

### 2.4. Microsphere Characterization

Optical microscopy (Keyence™ BZ-X810, Itasca, IL, USA) was employed to characterize the microspheres. Image acquisition and dimensional analysis were performed using BZ-X800 Viewer^®^ and BZ-X800 Analyzer^®^ software. Microsphere size distributions were analyzed by plotting microsphere counts versus the radius, from which the mean radius and standard deviation were calculated. This same approach was applied to determine the PDMS shell thickness of the liquid-core microspheres.

### 2.5. Sensor Design

#### 2.5.1. Microsphere Sensor Fabrication

**Figure 2:**
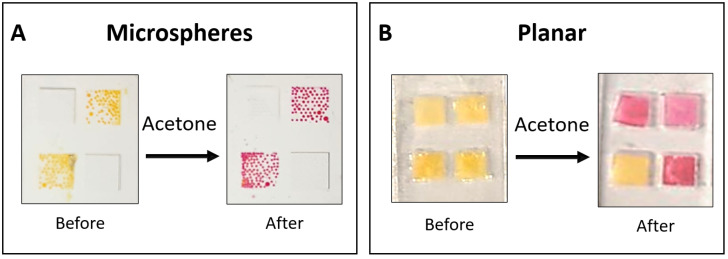
Sensor design. (**A**) Microsphere-based sensors were deposited onto a 4.00 mm × 4.00 mm transparent, micro-structured PET well and stabilized by a residual PVA layer. (**B**) A planar sensor was used for comparative time–response evaluation before and after exposure to acetone. The planar sensor was made of a 3D-printed mold and PDMS to produce square wells. Squared rayon paper was placed in each well, and the liquid sensing probe solution (HA-TB) was added to three wells, with TB solution added to the bottom left well as a control.

The liquid-core microspheres, which were produced according to the methodology detailed in Section 2.3, were deposited into 4.00 mm × 4.00 mm microstructured wells fabricated on a transparent polyethylene terephthalate (PET) substrate. The residual PVA outer layer from the fabrication process stabilized the microspheres, as they would collapse without it. Following drying in an acetone-free environment, the sensors were stored at 4 °C under a nitrogen atmosphere with 100% RH within sealed, aluminized Mylar bags. Figure 2A shows the assembled microsphere-based sensor before and after exposure to acetone.

Sensor stability was assessed over 2 weeks at two temperatures: 4 °C and 23.3 °C (room temperature, r.t.). For a preliminary evaluation of sensor stability, the following storage conditions were employed: sealed Mylar bags in a nitrogen atmosphere at 100% relative humidity (RH) for two weeks. The sensors demonstrated excellent stability at 4 °C and 23.3 °C throughout this 14-day storage period, as indicated by a negligible change in absorbance (in the green spectral region), as shown in Figure S7.

#### 2.5.2. Planar Sensor Fabrication

Planar sensors were used for comparative time–response evaluation before and after acetone exposure (Figure 2B). Three of the four wells contained the HA-TB sensing probe, which changed color from yellow to pink upon acetone exposure. The remaining well contained only TB, which served as a control, and showed no color change upon exposure to acetone due to the absence of hydroxylamine. The planar sensor incorporated a 4.00 mm × 4.00 mm square piece of grade 1281 high-purity cotton/rayon blend paper to hold the liquid sensing probe securely. This paper exhibited the following characteristics: basis weight 70 g/m^2^, caliper 0.38 mm, wicking rate 65 s/4 cm, and water absorption 60 mg/cm^2^. The PDMS housing was fabricated from two identical parts bonded together with PDMS. These parts were cast using a 3D-printed mold (Ultimaker 3D printer, PLA filament, New York, NY, USA). The internal mold walls measured 0.75 mm in height, while the square wells had a height of 0.47 mm. This design produced wells with a liquid capacity of 7.5 μL, of which ~3 μL contained the liquid sensing probe, including the embedded rayon paper [24].

Sensor stability was further evaluated. Figure S8 illustrates a freshly prepared sensor (top), a sensor stored at room temperature for one year (middle), and the same aged sensor after acetone exposure (bottom). As shown in the insets of Figures S1–S3, the planar sensor stored for one year retained its initial yellow color (indicating no degradation) and remained functional upon acetone exposure (pink color change). In contrast, the control inset (TB only, without HA) remained yellow after acetone exposure.

### 2.6. Detection Setup

The experimental setup for acetone detection is described in our previous work [24]. A custom sensing device was designed to study the colorimetric response of the sensors. The setup consisted of a complementary metal-oxide-semiconductor (CMOS) chip integrated with a white light-emitting diode (LED) source in an optical transmittance mode configuration. The CMOS camera, which was interfaced with a Raspberry Pi microcontroller (Raspberry Pi Foundation, Cambridge, UK), captured 10-s lapse images of the sensors during analyte exposure. These images were processed using a custom Python™ v.3.1.2 algorithm (Wilmington, DE, USA) to deconvolute the color data into red, green, and blue (RGB) intensity values [24]. Instead of direct camera calibration, the measured RGB values were referenced using ImageJ software (version 1.54g, Wayne Rasband and contributors, National Institutes of Health, USA), which served as the benchmark for color resolution. Formal calibration standards were not employed, as our measurements focused on the slope change (ΔA/Δt, absorbance vs. time). Color variation relative to baseline (initial RGB values) was quantified. The device components were housed within a polytetrafluoroethylene (PTFE) structure to ensure stability and alignment. Figure S9 illustrates the experimental design for analyzing simulated and real breath samples using microsphere-based sensors. The analysis consisted of a 5-min baseline exposure to humidified nitrogen (100% RH), followed by a 45-min exposure to the sample. Simulated breath samples were prepared in bags on the lab bench, while real breath samples were collected from human subjects in 40L balloons. The laboratory temperature was maintained at 23.3 °C. Simulated breath samples were processed individually in the laboratory. A pre-calibrated pneumatic pump, regulated by a precise voltage-controlled frequency inverter, was used to generate the specified gas ratios. The pump’s flow rate was calibrated against a standard 3 L volume of air with a known composition.

The sensor’s R, G, and B intensity values (*I*) were recorded concurrently as a function of exposure time and processed according to Equation (1), as follows:

(1) $$\Delta A=-log\left(\frac{I_{S}}{I_{R}}\right),$$

where *I_S_* is the intensity of the R, G, or B components within the sensing area of interest (e.g., HA-TB or TB area) at a given time, and *I_R_* is the intensity of the same R, G, or B component in a reference area (e.g., the center of the sensor without the sensing probe) captured at the same time. The changes in the sensor signal were analyzed as a function of different concentrations of acetone and carbon dioxide, thereby enabling the evaluation of sensor performance under different analyte conditions.

### 2.7. Samples

#### 2.7.1. Simulated Samples

To evaluate the sensor’s performance, simulated breath samples were prepared as follows: 4.02% CO_2_ and 16.10% O_2_ in a nitrogen balance (20 L, Matheson™ 1545 Watkins St., Phoenix, AZ, USA) was introduced into 40 L Tedlar^®^ bags to mimic a component of breath. To achieve a realistic 100% RH and approximate exhaled breath temperature (EBT, ~34.5 °C), 0.84 mL of deionized water was added to each bag at a room temperature of 23.3 °C, followed by heating to 40 °C for 10 min to ensure water saturation and temperature equilibration. Acetone samples of known concentrations were created by injecting certified standard gas (GASCO™, 1000 ppm *v*/*v* acetone in nitrogen) into the same 40 L Tedlar^®^ bags.

To evaluate CO_2_ as an interferent in acetone detection by the microsphere-based sensor, the gas mixture was systematically diluted to achieve final CO_2_ concentrations of 0.5%, 1%, 2%, 3%, and 4%, reflecting physiologically relevant levels. This data was compared to previously reported data for CO_2_ as an interferent using planar sensors [24].

#### 2.7.2. Human Subject Breath Samples

An informed consent form approved by the Institutional Review Board at Arizona State (IRB protocol number: STUDY00016374) was obtained from each subject before the study (*n* = 4). Breath samples were collected in Tedlar^®^ bags from the recruited participants; breath acetone concentrations were quantified using the microsphere-based sensor and compared with simultaneous GC-MS analysis of the same samples.

To collect human breath samples, subjects (*N* = 4) were instructed to continuously exhale into a mouthpiece-adapted 28″ diameter aluminum balloon for 10 min. This method ensured sufficient breath volume for acetone sensor evaluation and GC-MS.

### 2.8. Solid-Phase Microextraction (SPME) Coupled with GC-MS

#### 2.8.1. Modification of SPME Fibers

Solid-phase microextraction (SPME) fiber assemblies (Supelco, Sigma-Aldrich™, St. Louis, MO, USA) with a 65 μm polydimethylsiloxane/divinylbenzene (PDMS/DVB) coating on 24 Ga fused silica and a manual holder assembly were used. To enhance the sensitivity of acetone detection, the fibers were modified with *o*-(2,3,4,5,6-pentafluorobenzyl)hydroxylamine hydrochloride (PFBHA, Sigma-Aldrich™). The acetone derivatization chemical reaction for GC-MS analysis is depicted in Figure S12. Specifically, this derivatization process involved immersing the fibers in a septum-sealed vial containing 1 mL of PFBHA solution (prepared in HPLC-grade deionized water, Sigma-Aldrich™). The solution was stirred at room temperature for 10 min to allow for PFBHA adsorption onto the fibers.

#### 2.8.2. Measurement of Acetone in Breath and Simulated Samples

Acetone concentrations in breath and simulated samples were measured via GC-MS (Agilent 6890N Network GC System coupled with a 5973 Network Mass Selective Detector, Santa Clara, CA, USA). GC separation was performed using a 30 m × 0.25 mm i.d. with a 0.25 μm film of an HP-5MS UI capillary column (Agilent Technologies, Santa Clara, CA, USA), with a nonpolar phase capillary column coating made with (5%-phenyl)-methylpolysiloxane. Helium carrier gas at 60 kPa head pressure and 50 mL/min flow rate was employed. The injector temperature was maintained at 250 °C, and the detector temperature was 230 °C. The column temperature started at 70 °C for 2 min and was then programmed to 270 °C at a rate of 20 °C/min. With these settings, acetone and its oxime derivative had retention times of 6.62 and 5.54 min, respectively, within the 13-min chromatographic run. The mass spectrometer was operated in selected ion monitoring mode (SIM) at *m*/*z* of 100 and 340. The data collection was performed with MSD Chem Station Data Analysis Application Software (Agilent Technologies Inc., Santa Clara, CA, USA) running on a personal computer. The GC-MS was calibrated via on-column injections of gaseous acetone standards.

## 3. Results

### 3.1. Comparative Sensor Response

Experimentally, we have observed fast degradation (color change) of the sensing probe when prepared in a dry state (on cellulose paper or silica) and exposed to the atmosphere at room temperature within 24 h. This degradation was not observed when HA-TB was in solution (water, glycerol, methanol), likely because the solvent maintains a specific pH (4–6) environment that is essential for the stability of the sensing probe reagents. Therefore, we developed a sensing probe (HA-TB) stabilized within a polymeric matrix.

**Figure 3:**
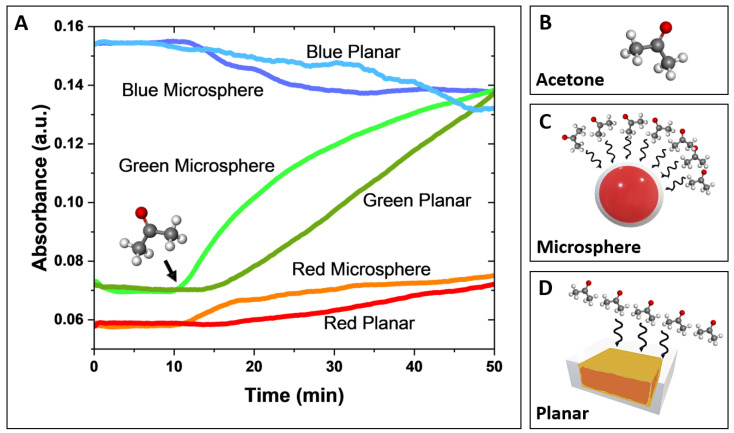
(**A**) Comparative response of microsphere and planar sensors to acetone. Changes in red, green, and blue absorbance were monitored upon exposure to ultra-pure nitrogen, followed by the injection of 300 ppm of acetone (indicated by the black arrow). Data were obtained using a white LED, CMOS detector, and Python imaging acquisition software, which were used to perform deconvolution of the red, green, and blue light components. (**B**) Molecular structure of acetone. (**C**,**D**) Schematic representation of the interaction (black wavy arrows) between gas acetone molecules and the microsphere-based sensor (**C**) and the planar sensor (**D**). The larger surface area of the microsphere-based sensor results in a greater number of interactions with acetone molecules.

A comparative analysis of the response time-resolved signal behavior, derived from deconvoluted red, green, and blue absorbance components, for both microsphere-based and planar sensors is presented in Figure 3A. In this experiment, both sensor designs were exposed to 300 ppm of acetone gas, delivered at a controlled flow rate of 0.75 L/min for 40 min. The 300 ppm acetone exposure lasted 40 min (the starting point is indicated by a black arrow in Figure 3A). Before the acetone exposure, a 10-min background reading was recorded while the sensors were exposed to nitrogen.

The green absorbance component (A_G_) exhibited the most significant signal changes, defined by its slope (ΔA_G_/Δt) for both the microsphere-based and planar sensors, with a noticeably larger response and greater sensitivity observed for the microsphere-based sensor. The red and blue absorbance components showed smaller changes (lower slopes), with less distinction in response between the two sensor types. In both sensor designs, green and red absorbance increased, while blue absorbance decreased, which is consistent with previously reported spectral response observations for HA-TB sensing probes [21,24].

Notably, the microsphere-based sensor demonstrated a significantly faster response time than the planar sensor. This improved performance is due to the 3D geometry of the microsphere-based sensor, which facilitates the efficient spherical diffusion of the analyte. In contrast, the planar sensor performance is limited by slower planar diffusion, resulting in reduced analyte transport and interaction with the sensor surface (Figure 3C,D). Further details of these molecular-level interactions of microsphere-based and planar sensors are shown in Figure S10. These findings suggest that the microsphere design leads to the faster and more sensitive detection of acetone due to its larger surface area and increased analyte interaction, making it a promising candidate for gas-phase sensing applications.

### 3.2. Microsphere Physical Characteristics

The microsphere size and compositional percentages were determined via the flow rates of the three solutions (sensing probe, PDMS, and PVA), which were optimized through various experimental combinations (see Table S1 and Figure S11, where we observed that higher flow rates resulted in larger microsphere sizes). Consequently, the flow rates were reduced to achieve smaller microspheres for an enhanced response (higher ratio area/volume). Furthermore, adjustments were made to obtain the thinnest possible shell while maintaining microsphere stability. Changes in flow rate directly affect the PDMS shell thickness, necessitating a compromise to achieve an optimal thickness. We aimed for an optimization between dehydration (if the PDMS membrane is too thin, moisture escapes from the core sensing probe, leading to microsphere dehydration) and response time (conversely, a thinner PDMS membrane allows for faster diffusion between the acetone and the sensing solution).

**Figure 4:**
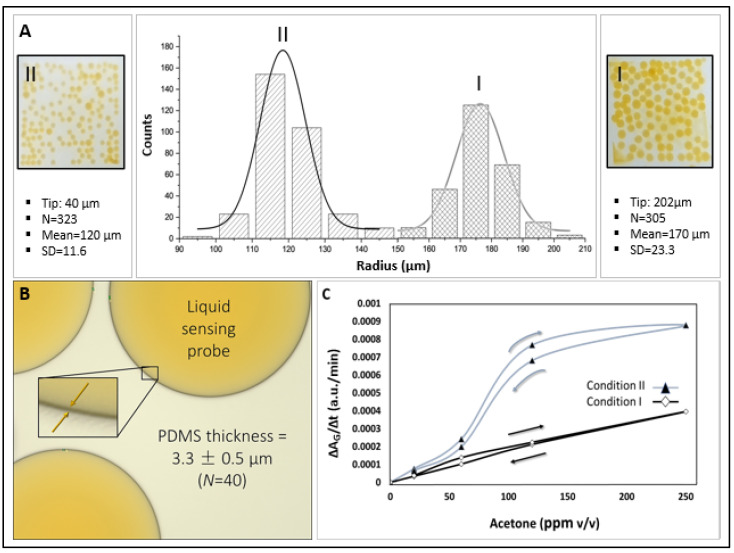
(**A**) Size distribution (microsphere count vs. radius) for microspheres fabricated under Conditions II and I (*N* = 323 and *N* = 305, respectively, Table 1). (**B**) Micrograph of representative microspheres highlighting the encapsulated liquid sensing probe. Inset: Magnified view showing the average PDMS membrane thickness. (**C**) Comparison of green absorbance sensitivity for microsphere sensors fabricated under Conditions I and II.

Figure 4A and Table 1 show the effect of fabrication parameters on microsphere size distribution. Size distribution curves (microsphere counts vs. radius) are presented for two sets of fabrication parameters, which result in microspheres of different sizes. Condition I, using a 202 µm tapered tip and liquid flow rates of 1000 µL/h (PVA), 250 µL/h (PDMS), and 70 µL/h (sensing probe), produced microspheres with a radius of 170 ± 23.3 µm. Condition II, with a 40 µm tapered tip and flow rates of 300 µL/h (PVA), 100 µL/h (PDMS), and 60 µL/h (HA-TB sensing probe), yielded smaller microspheres that measured 121 ± 11.6 µm in radius. Both sets of microspheres had a PDMS shell thickness of 3.3 ± 0.5 µm, *N* = 40 (Figure 4B). The resulting average microsphere densities in the PET sensor wells were 99 and 145 counts/well for conditions I and II, respectively.

The sensitivity of the microsphere sensors produced under both conditions was evaluated using known acetone concentration samples (5–250 ppm *v*/*v*) by monitoring the slope (ΔA_G_/Δt, as depicted in Figure 4C), which represents the green absorbance changes over time. Sensors fabricated under Condition II exhibited a higher sensitivity. This enhanced sensitivity can be attributed to two factors: first, the higher area/volume ratio of Condition II, which may have produced an increased diffusion of acetone, and second, the ratio of the outer PVA to sensing probe was lower for Condition II (300:60) than for Condition I (1000:70). The higher PVA content in Condition I likely contributed to a thicker PVA layer, which reduced the interaction efficiency between the sensing probe and acetone during acetone sensing experiments.

### 3.3. Microsphere Sensor Response to Acetone and Carbon Dioxide

**Figure 5:**
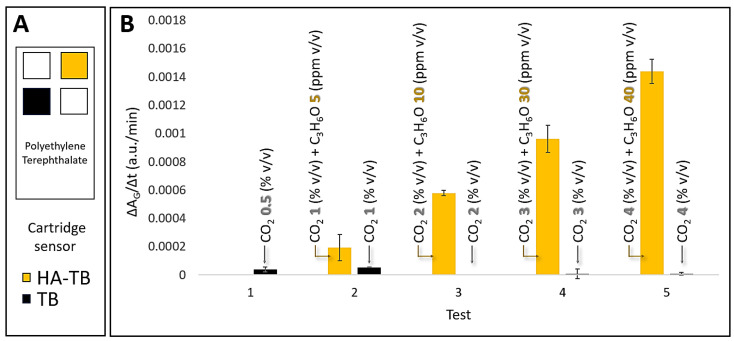
(**A**) Schematic representation of the PTE sensor cartridge detailing the precise location of the HA-TB and TB microsphere-based sensors within the cartridge. (**B**) Comparative response of HA-TB and TB microsphere sensors (*N* = 2). Green absorbance changes (a.u./min) were measured for varying concentrations of acetone (C_3_H_6_O) (0–40 ppm *v*/*v*) and carbon dioxide (CO_2_) (0–4% *v*/*v*) in a controlled environment of 100% RH. The data are presented as the rate of change in green absorbance (a.u./min), which corresponds to the slope of the raw sensor signal.

Our previous studies have identified that carbon dioxide (CO_2_) can interfere with acetone detection using the HA-TB sensing probe in a planar sensor and real samples. This is due to CO_2_’s ability to dissolve into the liquid probe (aqueous media) in the planar sensor, thereby altering the local pH and affecting the sensor’s response. To address this issue, we systematically evaluated the response of both HA-TB and TB microsphere sensors to varying concentrations of acetone and CO_2_ under controlled conditions. These experiments were conducted at 100% relative humidity, simulating realistic breath conditions. Figure 5A illustrates a schematic of the (polyethylene terephthalate, PET) sensor cartridge, highlighting the exact positions of the HA-TB and TB microsphere-based sensors inside the cartridge. Figure 5B demonstrates that the slope (ΔA_G_/Δt, green absorbance changes over time) of the HA-TB microsphere sensor remained unaffected by CO_2_ concentrations up to 4%, representing the upper limit typically found in human breath [24].

These results confirm the selectivity of the HA-TB microsphere sensor’s green absorbance response for acetone detection, with negligible interference from CO_2_. The significant differences in CO_2_ responsiveness between the HA-TB liquid sensing probe in planar sensors and the lack of responsiveness in microsphere-based sensors may be due to interactions or chemical reactions with the paper (Rayon) substrate of the planar sensor. Therefore, the green absorbance response of the microsphere-based sensor to acetone is optimal for minimizing CO_2_ interference, making it a more desirable option. Consequently, the signal processing algorithm for this sensor was further optimized (Section 3.4).

### 3.4. Continuous and Non-Invasive Acetone Monitoring with a Microsphere-Based Sensor

**Figure 6:**
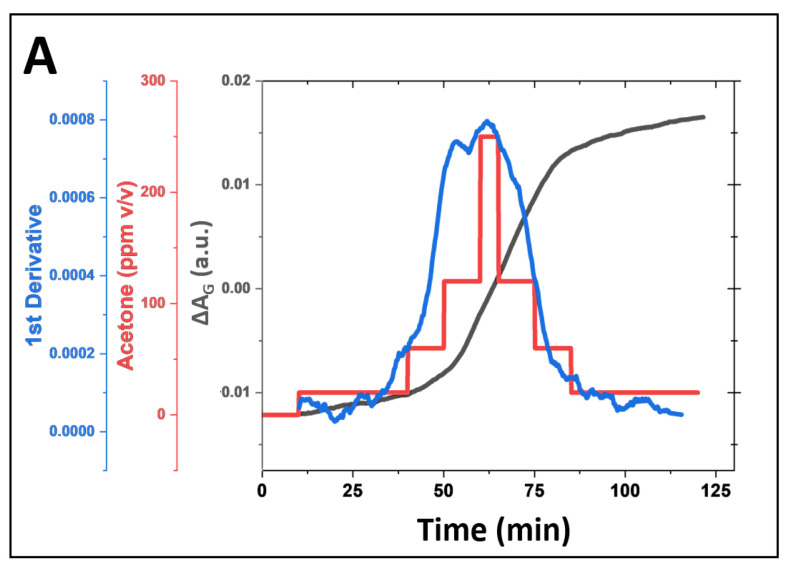

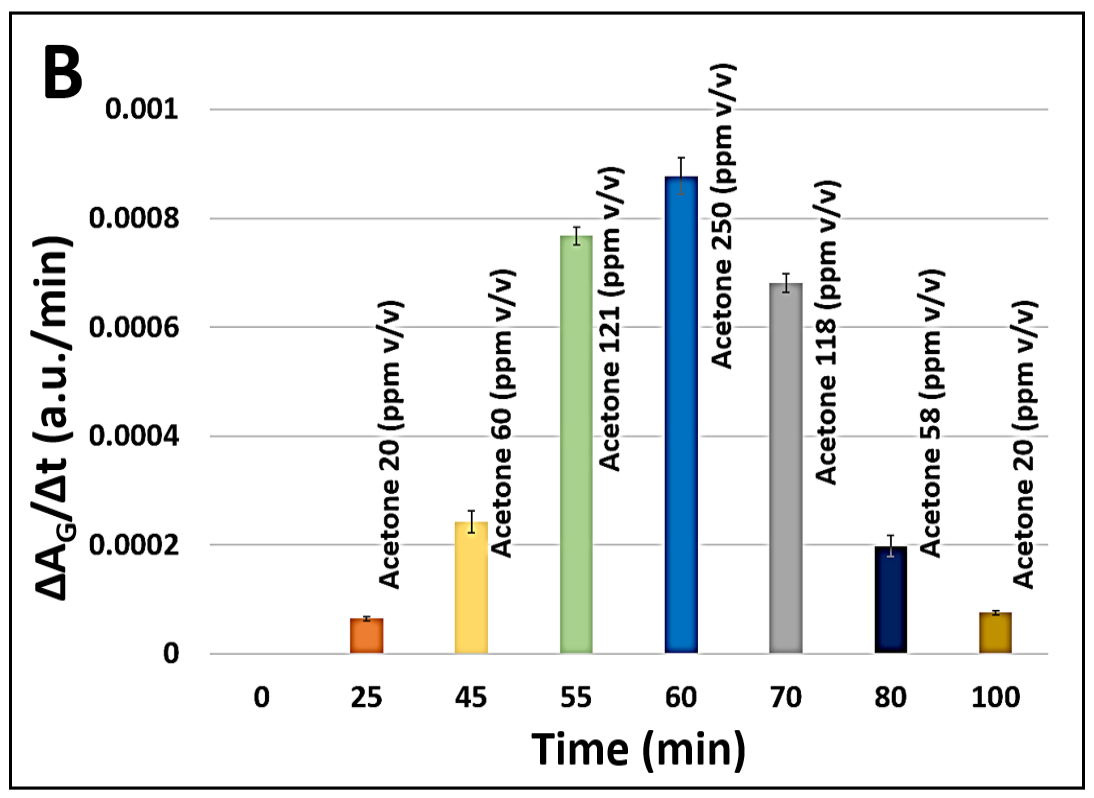
(**A**) Simulation of continuous acetone monitoring, including the sensor signal processing of microsphere sensor (180 µm radius, for green absorbance acquisition, black line), continuous HA-TB microsphere-based sensor response to increasing and decreasing acetone concentrations (red line), and the first derivative of the sensor response (blue line). (**B**) Equation (2) allows for real-time acetone concentration determination during continuous monitoring.

We investigated the feasibility of non-invasive acetone detection and continuous ketone monitoring (CKM) applications using the HA-TB microsphere-based sensor. Figure 6A (red line) presents the green absorbance response of the HA-TB microsphere sensor when exposed to acetone concentrations that were increased from 0 to 250 ppm and then decreased from 250 to 0 ppm to assess its performance across a relevant range of concentrations. Building on the previous work of Wang R., Forzani E. et al. [25], who modeled the response of colorimetric sensors, we analyzed the relationship between the absorbance change of the microsphere sensor and the gas concentration over time (Figure 6A, gray line), as described in the following equation:

(2) $$Acetone\;Concentration\;\left(t\right)=\frac{1}{k(∆A_{max}-∆A)}\frac{d\Delta A}{dt},$$

where ΔA is the absorbance change, ΔA_max_ represents the maximum absorbance change, and *k* is the calibration constant coefficient. According to Equation (2), the derivative of the sensor’s absorbance with respect to time is directly proportional to the analyte concentration. Based on this relationship, we calculated the first derivative of the microsphere sensor’s green absorbance response and compared it to the acetone concentration values used during exposure. Figure 6A (blue line) illustrates the sensor’s first derivative during exposure to increasing and decreasing acetone concentrations. Notably, the derivative closely follows the changes in concentration, confirming the predictive accuracy of Equation (2). This observation demonstrates that the first derivative of the sensor signal can provide real-time, instantaneous acetone concentration readings, making it suitable for applications that require continuous monitoring. Such applications may include non-invasive and needle-free methods for detecting acetone levels.

Moreover, our data indicate that the number of sensing probes stored within the microspheres is sufficient to sustain prolonged exposure to acetone. The minimal hysteresis, which shows that the first derivative values for the same acetone concentrations during the experiment’s ascending and descending portions matched, is proof of this. The lack of hysteresis confirms the sensor’s reliability and stability for continuous acetone monitoring. These findings suggest that this microsphere-based sensor, combined with Equation (2), offers a robust solution for real-time, non-invasive acetone monitoring in applications such as breath analysis or skin detection.

### 3.5. Microsphere Sensor Performance Evaluation: Reproducibility, Stability, and Accuracy

We evaluated the sensor responses to assess the reproducibility within and between the sensor batches. We stored the prepared sensors in a clean air environment (see Section 2.5) before testing and measured the response (slope = ΔA/Δt) of a fresh sensor. The coefficient of variability (CV), defined as the ratio between the sensor response average and the sensor response standard deviation for the same batch of sensors, was <5% on average, as shown in the error bars in Figure 5. This CV within the batch was acceptable for analytical standards.

The acetone microsphere sensor showed stability in the sensitive detection of acetone for extended periods when stored at appropriate temperatures. Similar to our previously published sensor [24], we found that the new liquid sensing microsphere sensor could maintain stability for at least 2 weeks when stored at 4 °C in an enclosed, clean, and humid environment with 100% RH (see Section 2.5).

**Figure 7:**
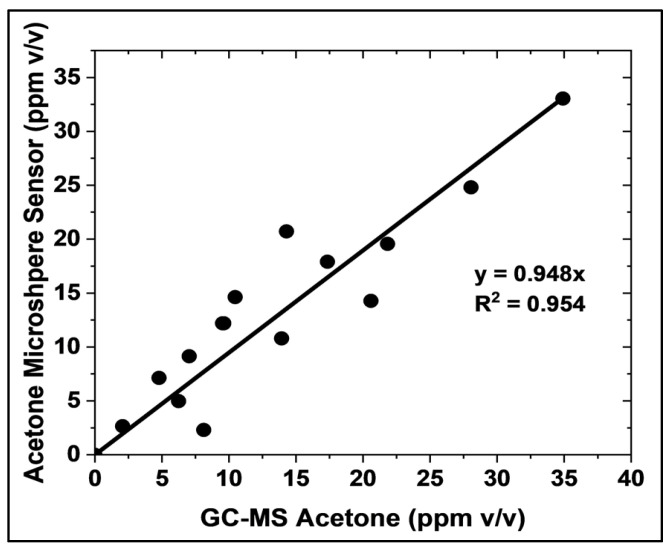
Comparison of acetone concentration measurements obtained using the HA-TB microsphere-based sensor and GC-MS (the gold standard method). Measurements were performed in 4.02% *v*/*v* CO_2_ and 100% relative humidity. The solid line depicts the linear regression (*N* = 15).

Calibration curves for the liquid-core microsphere and GC-MS methods are presented in Figures S13a and S13b, respectively. The liquid-core microsphere method yielded a Limit of Detection (LOD) of 0.189 ppm (189 ppb) and a Limit of Quantification (LOQ) of 0.572 ppm (572 ppb). In contrast, the GC-MS methodology exhibited a LOD of 0.328 ppm and a LOQ of 0.994 ppm. Based on these calibration curves, it can be estimated that the microsphere-based sensor exhibits a lower limit of detection (LOD) and lower limit of quantification (LOQ) ~57% better than the gold standard method (GC-MS). These results indicate a higher sensitivity for acetone detection in breath samples when employing the liquid-core microsphere method. The accuracy of the microsphere-based acetone sensor was evaluated using the same samples and by comparing its measurements with those obtained using GC-MS, which is the gold standard method, under realistic conditions (Figure 7). A strong correlation was observed between 2 and 35 ppm *v*/*v* acetone, with a linear fit of slope equal to 0.948, exhibiting an R-squared adjustment of 0.954. This correlation was established using 15 samples prepared with known acetone concentrations, 4.02% *v*/*v* CO_2_, and 100% relative humidity. The presence of CO_2_ and high humidity in these samples confirms the suitability of the microsphere sensor for point-of-care and non-invasive acetone measurement in gas-phase biological fluids. This finding demonstrates the strong agreement between the microsphere-based sensor and the established GC-MS method.

### 3.6. Microsphere Sensor for Continuous Acetone Monitoring in Type 1 Diabetes Patients

**Figure 8:**
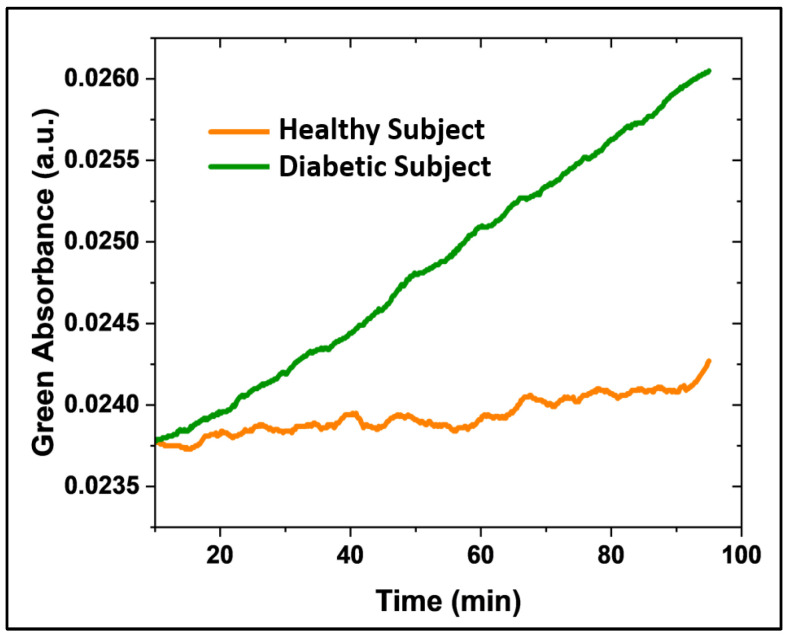
Continuous acetone monitoring (green absorbance acquisition) in a type 1 diabetic patient (green line, *N* = 2) and a healthy patient (orange line, *N* = 2) over time.

Preliminary results from the breath samples of Type 1 diabetic patients and healthy subjects demonstrated the sensor’s capacity to differentiate between varying acetone levels in real human breath, as further evaluated in a small cohort of healthy (*N* = 2) and diabetic (*N* = 2) individuals. Each patient was fitted with a clean mask connected to a 40 L aluminum balloon, where a breath sample was collected over 10 min. The balloon was subsequently connected to the detection system (Figure S9) to record the delta absorbance over 45 min (with an adjusted flow rate) after 5 min of baseline collection (Nitrogen). A CMOS camera controlled by a commercial Raspberry Pi system captured images every 10 s. This capture frequency, programmed in Python, provided sufficient temporal resolution for detecting the colorimetric changes. The continuous acetone detection (green absorbance) in type 1 diabetic patients (green line) and healthy subjects (orange line) over time is shown in Figure 8. The sensor registered a greater slope in absorbance over time (ΔA/Δt) for the diabetic patient compared to the healthy subject.

While a more comprehensive clinical validation with greater statistical analysis is necessary for the clinical application of this sensor, here, we aimed to assess its performance with real breath samples and demonstrate the experimental feasibility of distinguishing acetone concentrations between healthy and diabetic individuals using this novel methodology. Thus, this preliminary study establishes the sensor’s ability to discriminate acetone concentrations in type 1 diabetic patients. Ongoing research focuses on enhancing sensor sensitivity and conducting a quantitative evaluation.

## 4. Discussion

This study introduces a novel liquid-core microsphere-based colorimetric sensor as an effective tool for the sensitive and selective detection of acetone in both simulated and real human breath samples, *as well as potentially in skin samples*, that utilize HA and TB as sensing reagents. The preliminary results indicate the successful development of this promising sensor for the non-invasive and continuous monitoring of acetone levels, demonstrating excellent accuracy and stability. This performance was validated against the gold-standard GC-MS method, underscoring its potential for point-of-care applications in metabolic health management.

Innovation in Sensor Design: The liquid-core microsphere design effectively overcame the limitations of previous planar sensors. The three-dimensional structure of the microspheres provided a larger surface area for interaction with acetone molecules, resulting in a faster and more sensitive response. As shown in Figure 3, the microsphere-based sensor exhibited more pronounced response slopes (absorbance change over time), particularly in the green component (ΔA_G_), compared to the planar sensor when exposed to the same acetone concentration for the same time (note: the sensing probe volume is ~3 µL). Furthermore, encapsulating the sensing probe (HA-TB) within a PDMS shell resolved an important stability problem. The rapid degradation observed in sensing probes in a dry state (on cellulose paper or silica) did not occur in the microspheres, where the liquid core maintained a specific pH environment (4–6) that is essential for reagent stability. This innovation enabled a prolonged sensor lifetime of around 2 weeks when stored at 4 °C and 23 °C under 100% RH (demonstrated so far).

Precise Control of Sensor Characteristics: The developed fabrication process enabled precise control over microsphere size and PDMS shell thickness, which are essential factors for optimizing sensor response and sensitivity. As detailed in Table 1 and Figure 4, smaller microspheres exhibited higher sensitivity due to a greater surface area-to-volume ratio and a thinner residual PVA layer after water washes, thereby facilitating more efficient interactions between the sensing probe and acetone.

Selectivity and Analytical Performance: A significant achievement of this study was the elimination of CO_2_ interference in acetone detection. Unlike previous planar sensors, the microsphere-based sensor showed no significant response to CO_2_ at concentrations up to 4%—the typical upper limit found in human breath—as illustrated in Figure 5. This high sensitivity, combined with a strong correlation with GC-MS (R^2^ = 0.954) within the relevant range of 2–35 ppm (Figure 7), confirms the accuracy and reliability of the sensor for clinical applications, further validating its performance.

Continuous Monitoring and Real-Time Applications: Another innovative aspect of this study was the development of a signal processing algorithm that enabled the real-time determination of acetone concentration. As illustrated in Figure 6, the first derivative of the sensor response closely tracked changes in acetone concentration, thereby validating Equation (2) as an accurate predictive model. This ability to provide instantaneous concentration readings makes the sensor ideal for continuous monitoring applications.

The minimal hysteresis observed in the sensor’s response during increasing and decreasing acetone concentration cycles confirmed its stability and reliability for prolonged monitoring. This also demonstrates that the sensing probe volume stored within the microspheres is enough to extend acetone exposures without compromising performance.

## 5. Conclusions

The liquid-core microsphere-based colorimetric sensor developed in this study represents a significant improvement in acetone sensing for future non-invasive and continuous acetone monitoring. It has high sensitivity, selectivity against CO_2_ interference, and stability, while also providing real-time measurements for the promising and practical management of metabolic disorders.

## Figures and Tables

**Table 1:** Capsule dimensions under varying fabrication conditions.

Condition	Tapered Tip Diameter (µm)	Liquid Phase Flowrate (µL h^−1^)			Capsule Diameter (µm)	Capsule Membrane Thickness (µm)
		Outer	Middle	Inner		
I	202	1000	250	70	340.8	3.3 ± 0.5
II	40	300	100	60	241.8	

## Data Availability

Data is contained within the article.

## Supplementary Materials

The following supporting information can be downloaded at https://www.mdpi.com/article/10.3390/bios15070429/s1: Figure S1: Colorimetric acetone sensor reaction. Figure S2: Schematic of the microfluidic device on a glass microscope slide illustrating capillary dimensions and fluid inlets/outlets. PVA and PDMS solutions enter at the top ends, the sensing probe solution enters from the left, and microspheres are collected from the right. Figure S3: Microscopic image showing the microsphere formation region between the conical and flat cylindrical capillaries. Figure S4: Representative microsphere size using different devices with comparable dimensions. Figure S5: Experimental setup for microsphere fabrication showing the microscope and the three pumps for the sensing solution, PDMS, and PVA. Figure S6: Microsphere fabrication visualized using microscopy. Figure S7: Microsphere-based sensor stability test. Figure S8: Sensor stability: Freshly prepared sensor (top), sensor after one year at room temperature (middle), and sensor after acetone exposure (bottom). Figure S9: Experimental design for the analysis of breath samples using microsphere sensors. Figure S10: Planar (left) and microsphere-based (right) sensor designs. The molecular structures are not shown to scale. Figure S11: Capsule diameter (μm) vs. condition (increasing liquid phase flow rates). As the flow rates of the liquid phases increase, a larger diameter is observed in the microspheres. Figure S12: Acetone derivatization reaction for GC-MS analysis. Figure S13: (a) Calibration curve of microsphere-based sensor for acetone detection. (b) Calibration curve of GC-MS. The acetone concentration on the x-axis corresponds to samples obtained via the dilution of analytical grade calibration gas. Table S1: Flow rate variation for different layers (mL/h) vs. capsule rate and diameter (*n* = 30). Reference [26] is cited in Supplementary Materials.

## Abbreviations

The following abbreviations are used in this manuscript:

AcAc	Acetoacetate
% *v*/*v*	Percent Volume per Volume
°C	Celsius Degree
µL/h	Microliter per Hour
µm	Micrometer
a.u.	Arbitrary Units
B	Blue
BHB	β-Hydroxybutyrate
*c*	Constant = Initial Absorbance at Time Zero
C_3_H_6_O	Acetone
CMOS	Complementary Metal Oxide Semiconductor
CO_2_	Carbon Dioxide
CV	Coefficient of Variation
EBT	Exhaled Breath Temperature
G	Green
g/min	Grams per Minute
GC-MS	Gas Chromatography–Mass Spectrometry
HA	Hydroxylamine Sulfate
HA-TB	Sensing Probe (Hydroxylamine Sulfate and Thymol Blue)
HPLC	High-Performance Liquid Chromatography
*I*	Intensity
*I_R_*	Intensity of the R, G, or B components in the Reference Area
IRB	Institutional Review Board
*I_s_*	Intensity of the R, G, or B components in the Sensing Area
*k*	Reaction Rate Coefficient
LED	Light-Emitting Diode
LOD	Limit of Detection
LOQ	Limit of Quantification
min	Minutes
mL	Milliliter
MSD	Mass Selective Detector
*N*	Number
PDMS	Polydimethylsiloxane
PET	Polyethylene Terephthalate
PFBHA	*o*-2,3,4,5,6-(Pentafluorobenzyl)Hydroxylamine Hydrochloride
ppm	Part-Per-Million
PTFE	Polytetrafluoroethylene
PVA	Polyvinyl Alcohol
QR	Quick Response (A Type of Two-Dimensional Barcode)
R	Red
r.t.	Room Temperature
RGB	Red–Green–Blue
RH	Relative Humidity
SIFT-MS	Selected Ion Flow Tube Mass Spectrometry
SIM	Selected Ion Monitoring Mode
SPME	Solid Phase Microextraction
TB	Thymol Blue
ΔA	Absorbance Change (Final Absorbance − Initial Absorbance)
ΔAmax	Maximum Absorbance Change
ΔG/G0	Green Absorbance of the Sample Related to the Reference
