# Supplementary material for: A Microsphere-Based Sensor for Point-of-Care and Non-Invasive Acetone Detection
Source: Biosensors (Basel). 2025 Jul 3;15(7):429. doi: 10.3390/bios15070429 (PMC12293801; doi:10.3390/bios15070429)
Supplement: Supplementary file 1 [file biosensors-15-00429-s001.zip › biosensors-3545399-supplementary.pdf]

Supporting Information

A microsphere-based sensor for point-of-care and non-invasive acetone detection

Oscar Osorio Perez [1,2,3,†], Ngan Anh Nguyen [1,2,3,†], Landon Denham [1,2,3], Asher Hendricks [1,2], Rodrigo E. Dominguez [1,2,3], Eun Ju Jeong [1,2,3], Marcio S. Carvalho [4], Mateus Lima [4], Jarrett Eshima [5], Nanxi Yu [2], Barbara Smith [5], Shaopeng Wang [2,5], Doina Kulick [6,*], and Erica Forzani [1,2,3,*]

1 School of Engineering for Matter, Transport and Energy, Arizona State University, Tempe, AZ 85287, U.S.A.; oosoriop@asu.edu (O.O.P.); annguye6@asu.edu (N.A.N.); landondenham@asu.edu (L.D.); ajpete20@asu.edu (A.H.); ejeong5@asu.edu (E.J.J.)

2 Center for Bioelectronics and Biosensors, Biodesign Institute, Arizona State University, 1001 S McAllister Ave., Tempe, AZ 85281, U.S.A.; nanxiyu@asu.edu (N.Y.); shaopeng.wang@asu.edu (S.W.)

3 Medical Devices and Methods Laboratory, Health Futures Center, Arizona State University, 6161 E. Mayo Blvd., Phoenix, AZ 85054, U.S.A.; redoming@asu.edu (R.E.D.)

4 Department of Mechanical Engineering, Pontifícia Universidade Catolica do Rio de Janeiro, Prédio Pe. Laércio Dias de Moura - R. Marquês de São Vicente, 225 - 6º andar – Gávea Rio de Janeiro, Brazil.; msc@puc-rio.br (M.S.C.); mlima@lmmp.mec.puc-rio.br (M.L.).

5 School of Biological and Health Systems Engineering, G Wing, E. Tyler Mall, Tempe, AZ 85281, U.S.A.; jeshima@asu.edu (J.E.); barbarasmith@asu.edu (B.S.).

6 Mayo Clinic Arizona, 13208 E. Shea Blvd, Scottsdale, AZ 85259.

† These authors contributed equally to this work.

* Correspondence: kulick.mayo@mayo.edu (D. K.), eforzani@asu.edu (E. F.)

Table of Contents

Figure S1: Colorimetric acetone sensor reaction.

Figure S2: Schematic of the microfluidic device on a glass microscope slide.

Figure S3: Microscopic image showing the microsphere formation region between the conical and flat cylindrical capillaries.

Figure S4: Representative microsphere size using different devices with comparable dimensions.

Figure S5: Experimental setup for microsphere fabrication showing the microscope and the three pumps for the sensing solution, PDMS, and PVA.

Figure S6: Microsphere fabrication visualized using microscopy.

Figure S7: Microsphere-based sensor stability test.

Figure S8: Sensor stability: Freshly prepared sensor (top), sensor after one year at room temperature (middle), and sensor after acetone exposure (bottom).

Figure S9: Experimental design for the analysis of breath samples using microsphere sensors.

Figure S10: Planar (left) and microsphere-based (right) sensor designs.

Figure S11: Capsule diameter (μm) vs. condition (increasing liquid phase flow rates).

Figure S12: Acetone derivatization reaction for GC-MS analysis.

Figure S13: a) Calibration curve of microsphere-based sensor for acetone detection. **b)** Calibration curve of GC-MS.

Table S1. Flow rate variation for different layers (mL/h) vs. capsule rate and diameter (n = 30).

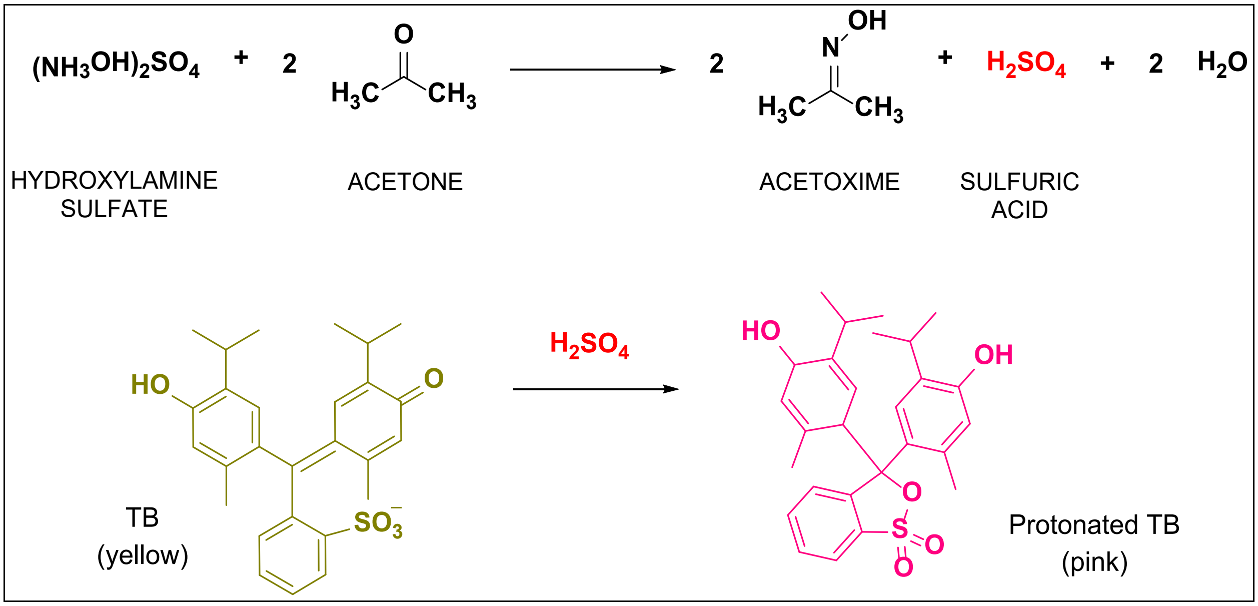

Figure S1. Colorimetric acetone sensor reaction.

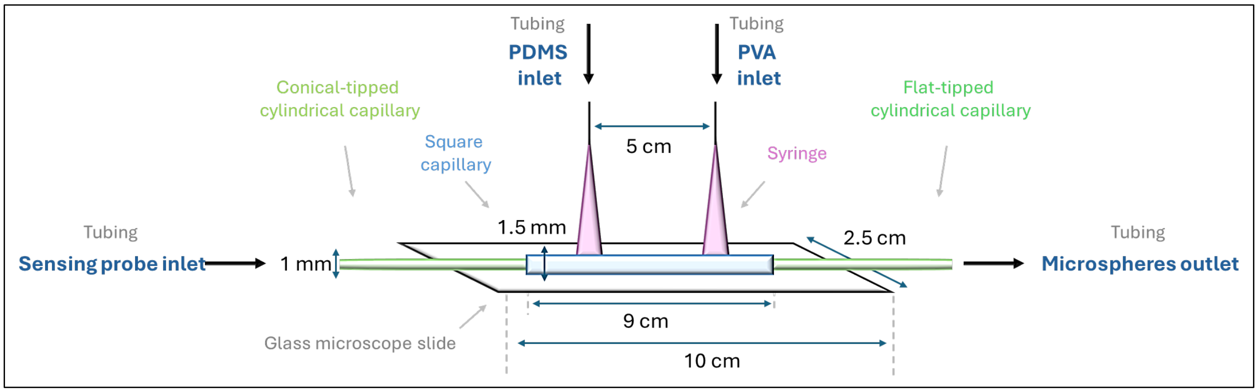

Figure S2. Schematic of the microfluidic device on a glass microscope slide illustrating capillary dimensions and fluid inlets/outlets. PVA and PDMS solutions enter at the top ends, the sensing probe solution enters from the left, and microspheres are collected from the right.

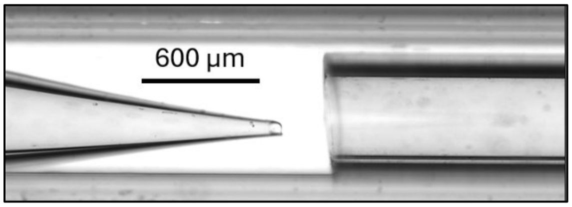

Figure S3. Microscopic image showing the microsphere formation region between the conical and flat cylindrical capillaries.

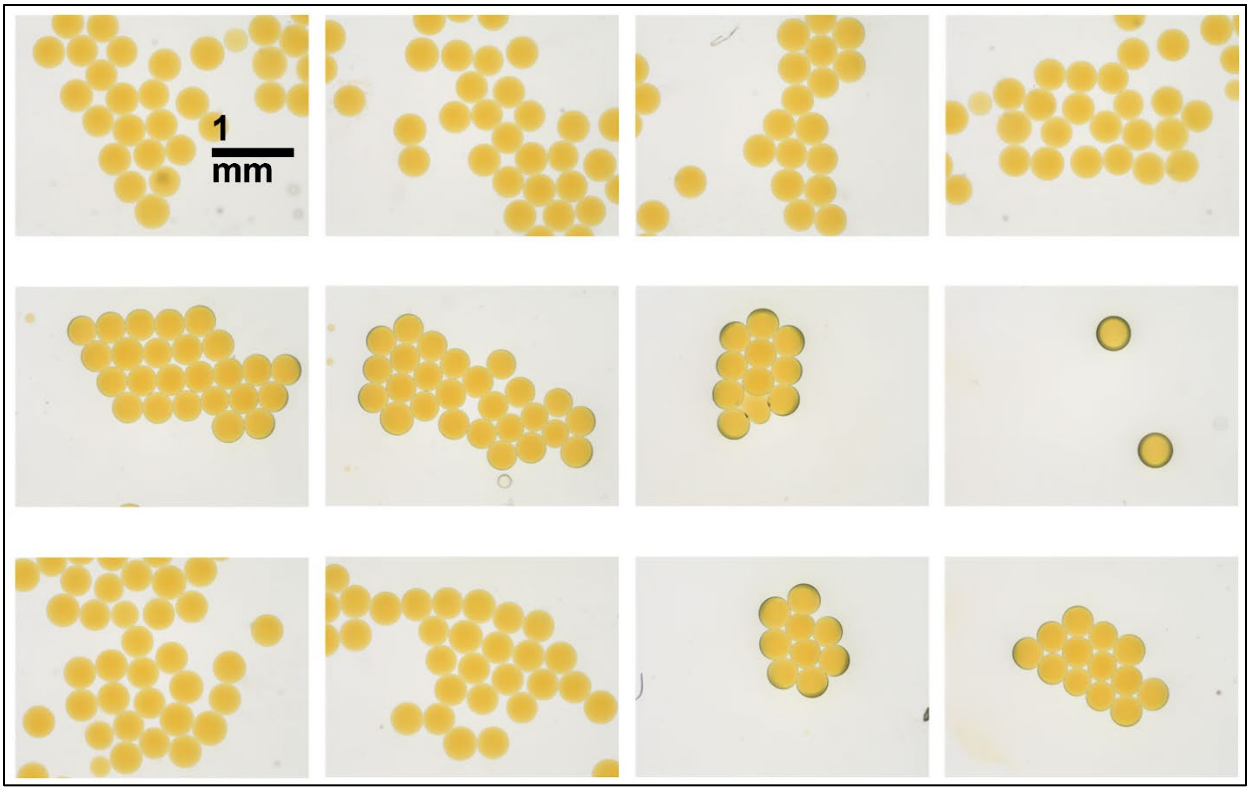

Figure S4. Representative microsphere size using different devices with comparable dimensions.

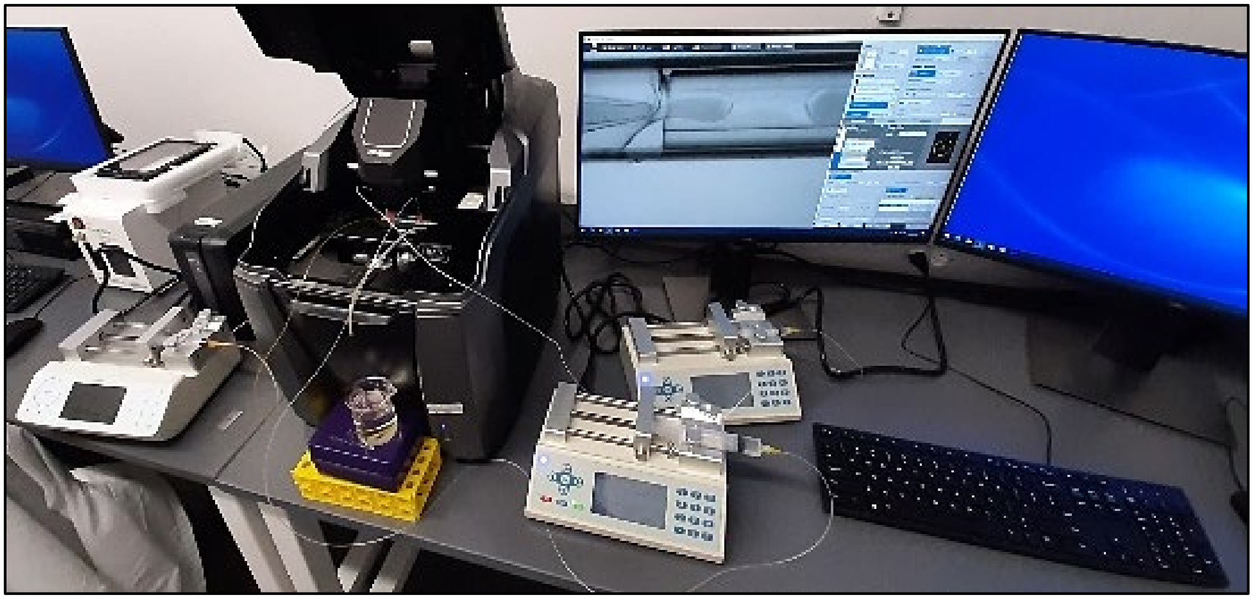

Figure S5. Experimental setup for microsphere fabrication showing the microscope and the three pumps for the sensing solution, PDMS, and PVA.

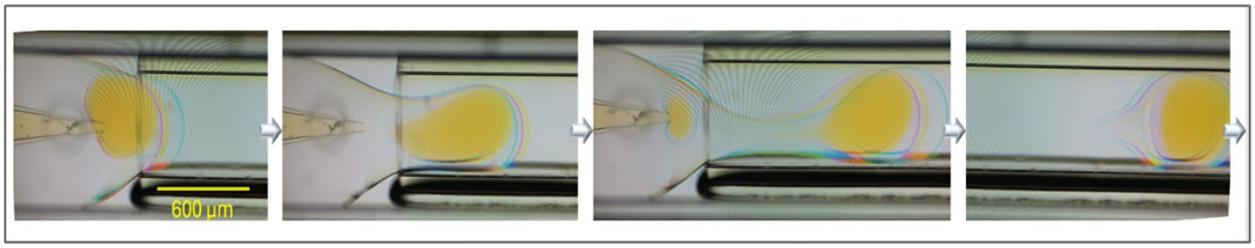

Figure S6. Microsphere fabrication visualized using microscopy.

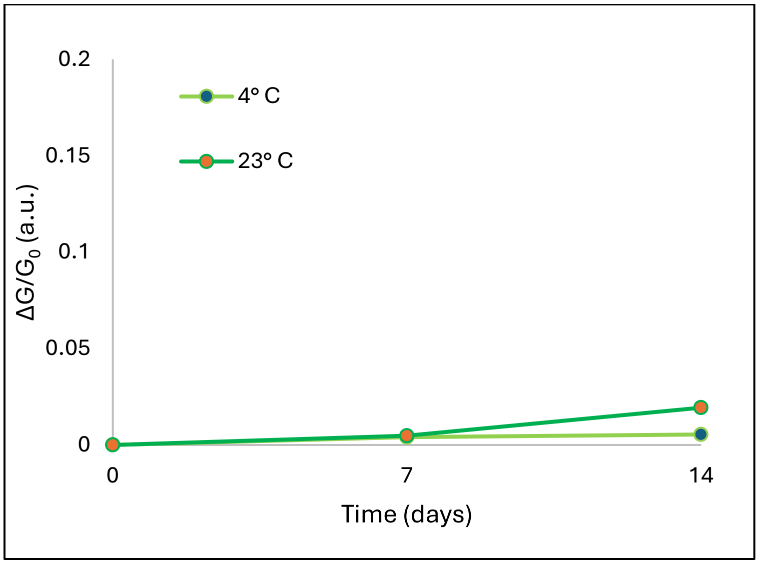

Figure S7. Microsphere-based sensor stability test.

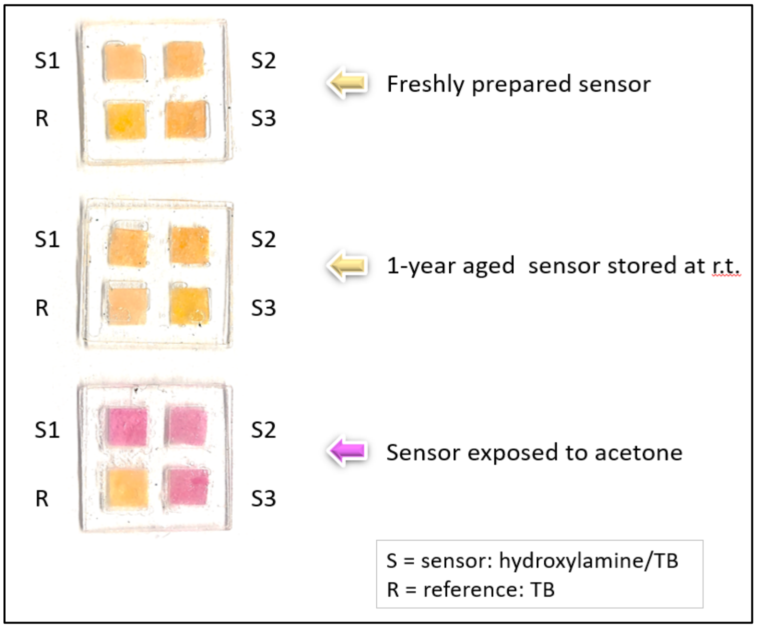

Figure S8. Sensor stability: Freshly prepared sensor (top), sensor after one year at room temperature (middle), and sensor after acetone exposure (bottom).

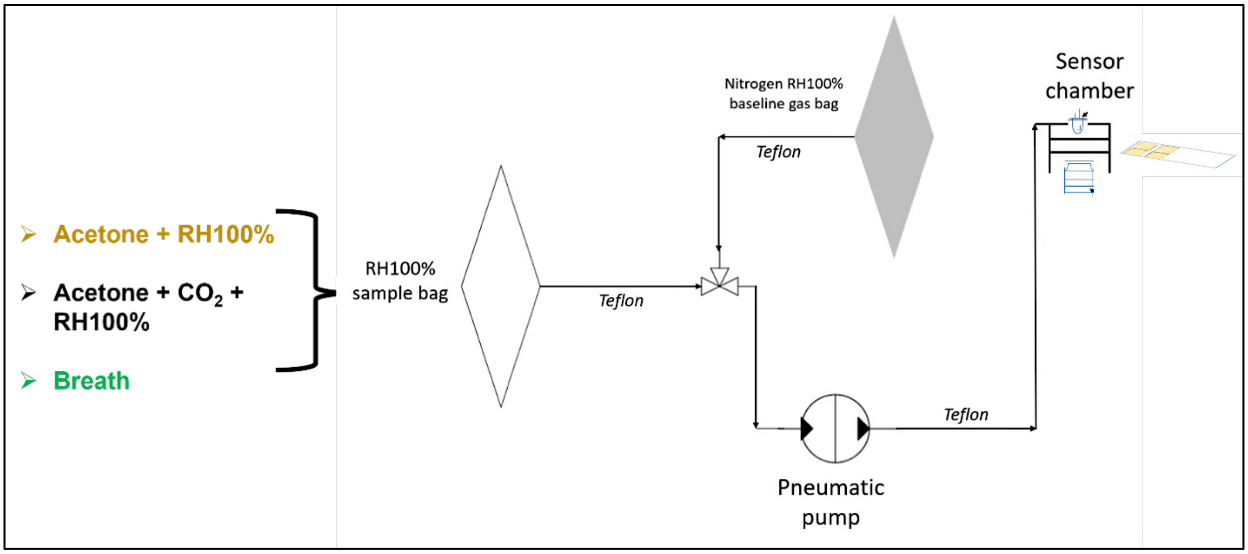

Figure S9. Experimental design for the analysis of breath samples using microsphere sensors.

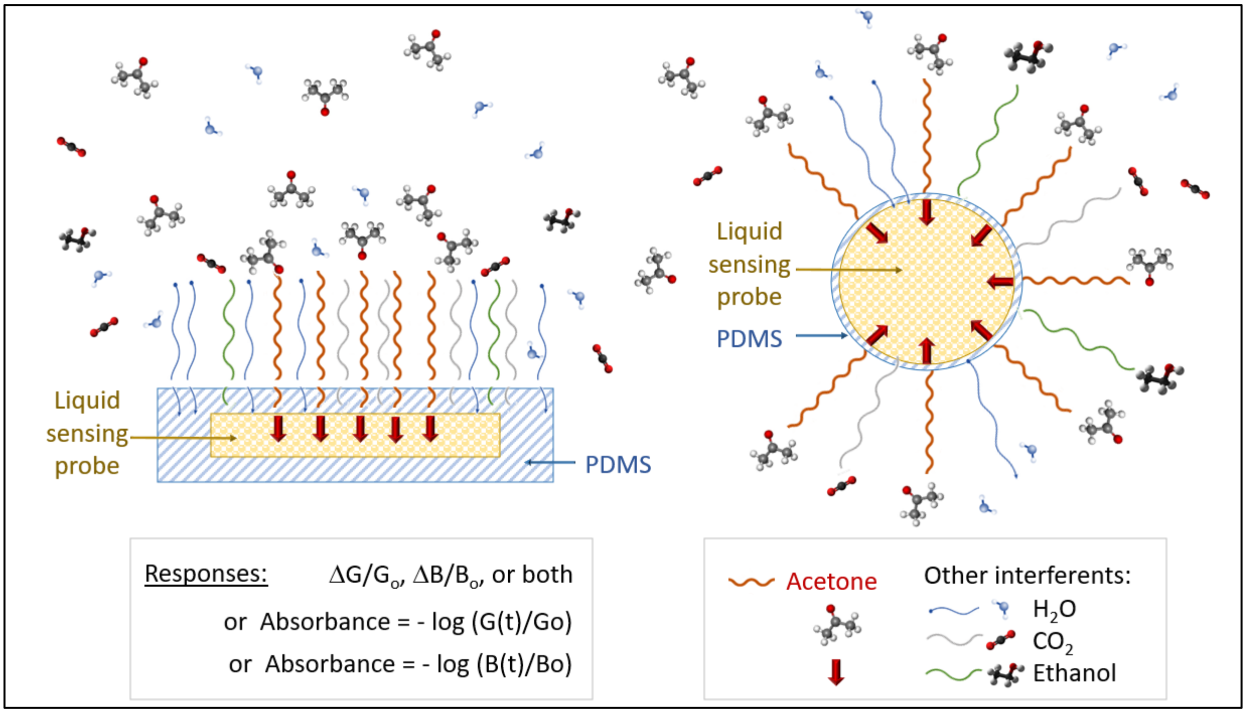

Figure S10. Planar (left) and microsphere-based (right) sensor designs. The molecular structures are not shown to scale.

Table S1. Flow rate variation for different layers (mL/h) vs. capsule rate and diameter (n = 30).

Condition	PVA	PDMS	HAS-TB	Capsule rate	Diameter (µm)	
	(µl/h)	(µl/h)	(µl/h)	(number /min)	Average (*N*=30)	SD (*N*=30)
**1**	200	100	70	15	379.00	23.34
**2**	300	100	70	16	371.00	11.55
**3**	450	100	70	19	421.14	20.00
**4**	600	100	70	19	390.00	29.04
**5**	1000	250	230	40	421.00	19.99
**6**	1300	250	230	47	481.42	8.75
**7**	1600	250	270	59	449.00	14.27
**8**	1800	250	400	62	515.00	15.51
**9**	2000	300	450	73	471.00	10.15
**10**	2200	500	650	105	481.00	8.76

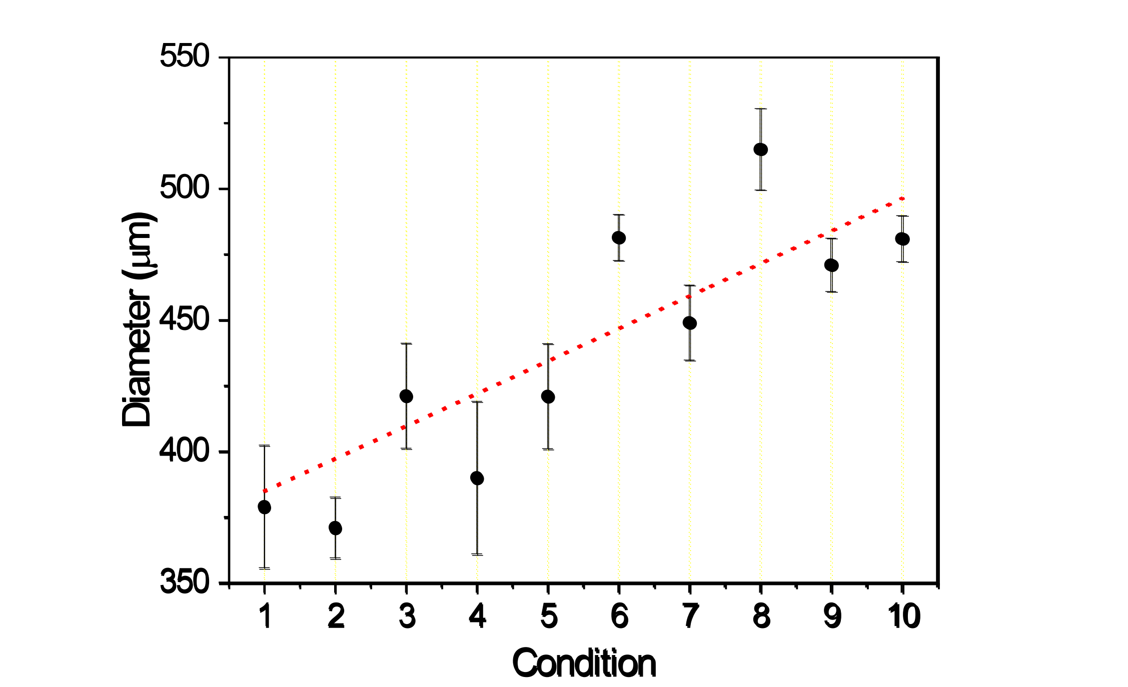

Figure S11. Capsule diameter (µm) vs. condition (increasing liquid phase flow rates). As the flow rates of the liquid phases increase, a larger diameter is observed in the microspheres.

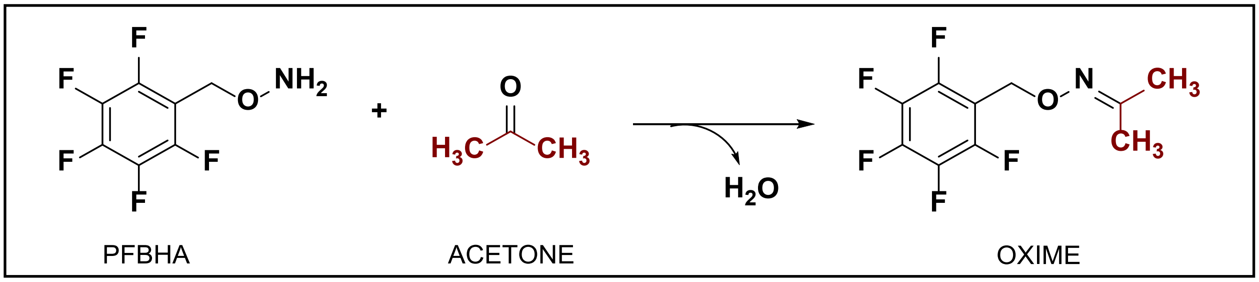

Figure S12. Acetone derivatization reaction for GC-MS analysis.

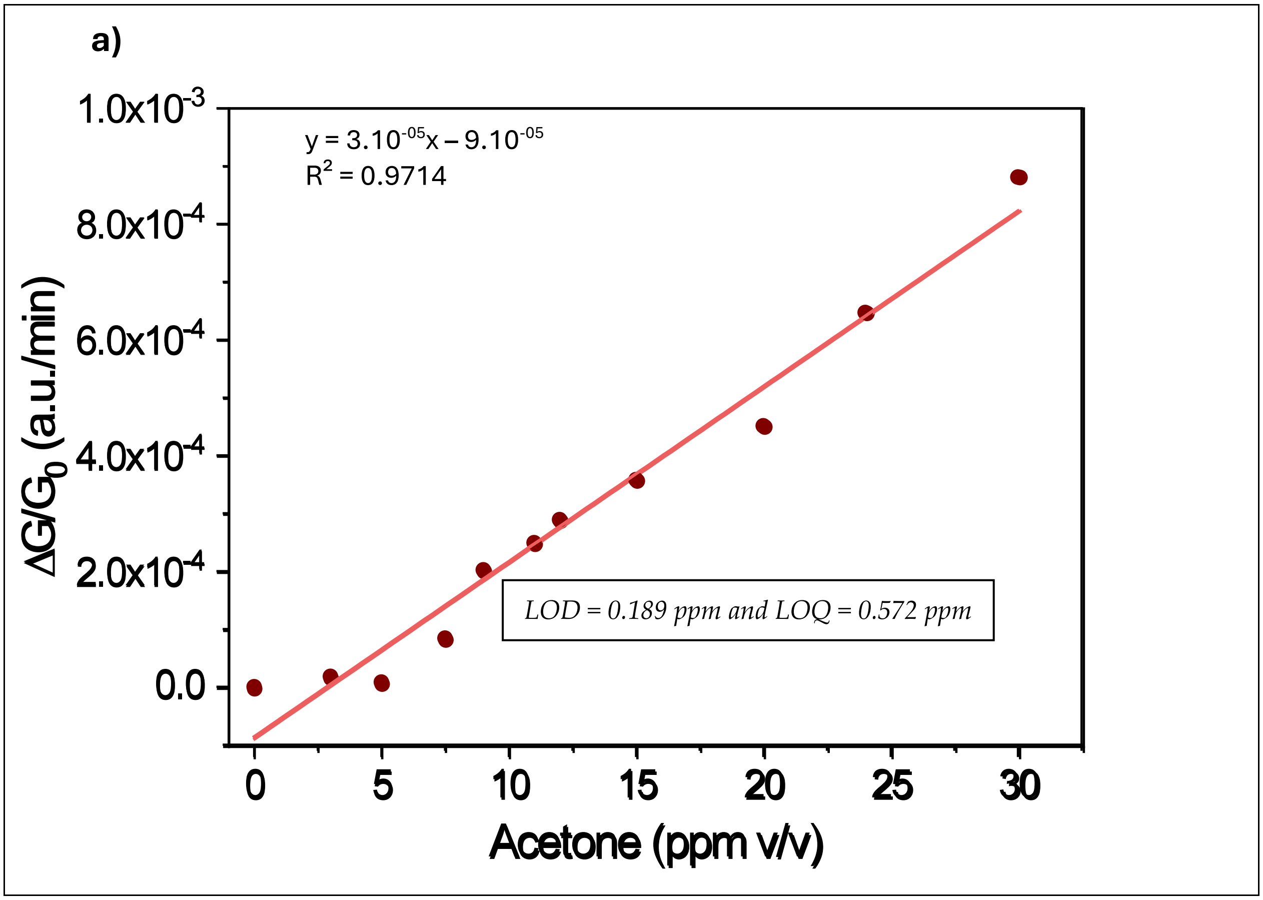

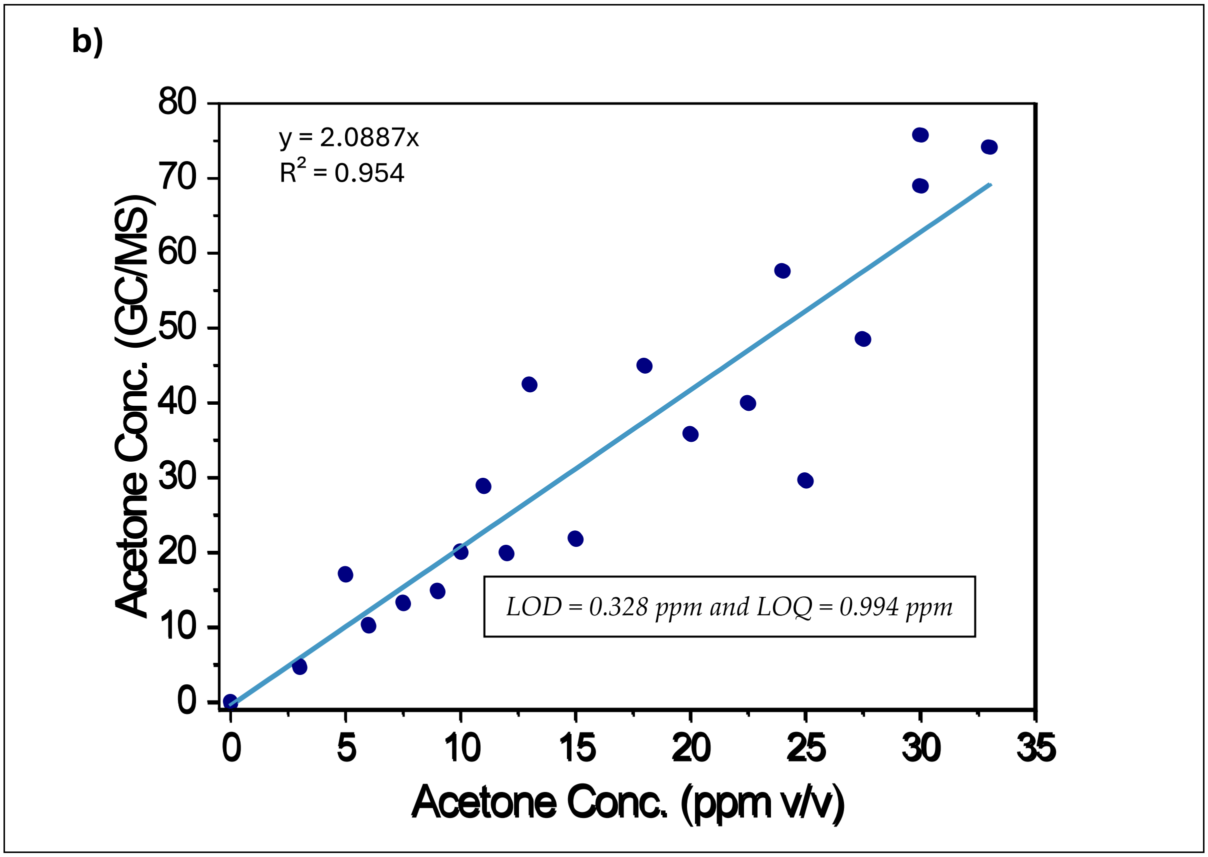

Figure S13. a) Calibration curve of microsphere-based sensor for acetone detection. **b)** Calibration curve of GC-MS. The acetone concentration on the x-axis corresponds to samples obtained via the dilution of analytical grade calibration gas.

The Limit of Detection (LOD) and Limit of Quantification (LOQ) were determined using the following equations:

$$\mathrm{LOD} = 3.3\,\frac{\mathrm{s_y}}{\mathrm{S}}$$

$$\mathrm{LOQ} = 10\,\frac{\mathrm{s_y}}{\mathrm{S}}$$

where $\mathbf{s_y}$ represents the standard deviation of the blank response (or background noise), which is obtained by measuring the signal of the blank a statistically significant number of times, and **S** represents the analytical sensitivity, which is estimated from the slope of the calibration curve. From the calibration curve shown in **Figure 13a**, we determined a standard error ($\mathbf{s_y}$) of 1.73×10^{-6}

and a slope (S) of 3.03×10^{-5}, yielding a Limit of Detection (LOD) of 0.189 ppm (equivalent to 189 ppb) and a Limit of Quantification (LOQ) of 0.572 ppm (equivalent to 572 ppb).

Based on these calibration curves, it can be estimated that the microsphere-based sensor exhibits a LOD and LOQ ~ 57% better (lower) than the gold standard method (GC-MS).[1]

References

[1] Dolan, J. (2021). Chromatographic measurements, part 5: Determining lod and loq based on the calibration curves. *Separation Science (9 Feb 2021). https://www. sepscience. com/hplc-solutions-126-chromatographic-measurementspart-5-determining-lod-and-loq-based-on-the-calibration-curve*.
